# Coexistence of intrathyroid thymic carcinoma and papillary thyroid carcinoma: a case report and literature review

**DOI:** 10.3389/fonc.2024.1394020

**Published:** 2024-05-03

**Authors:** Maryam Vajihinejad, Ali Ataei, Mohammad Pashmchi, Ali Aledavoud, Vahid Zand, Mohammad Ali Broomand, Mohammad Mohammadi, Niloofar Zare Reshkuiyeh

**Affiliations:** ^1^ Department of Pathology, Shahid Sadoughi University of Medical Sciences, Yazd, Iran; ^2^ School of Medicine, Bam University of Medical Sciences, Bam, Iran; ^3^ School of Medicine, Shahid Sadoughi University of Medical Sciences, Yazd, Iran; ^4^ School of Medicine, Kerman University of Medical Sciences, Kerman, Iran; ^5^ Department of Otolaryngology-Head and Neck Surgery, Otorhinolaryngology Research Center, Shahid Sadoughi University of Medical Sciences, Yazd, Iran; ^6^ Department of Clinical Oncology, Shahid Sadoughi University of Medical Sciences, Yazd, Iran

**Keywords:** intrathyroid thymic carcinoma, papillary thyroid carcinoma, thyroid, oncology, radiotherapy

## Abstract

**Background:**

Intrathyroid thymic carcinoma (ITTC) is a rare neoplasm of the thyroid, which accounts for less than 0.15% of all thyroid malignancies. The coexistence of ITTC and papillary thyroid carcinoma (PTC) is an extremely rare condition reported only in a limited number of cases.

**Case summary:**

A 26-year-old female presented with a growing neck mass, hoarseness, and dysphagia over four months. Ultrasonography revealed that the entire left lobe and the isthmus of the thyroid were replaced with a hypoechoic mass. Moreover, it revealed two hypoechoic nodules in the right thyroid. The patient underwent a total thyroidectomy and paratracheal lymph node dissection. Histopathological examinations revealed the coexistence of ITTC and PTC in the same thyroid. In immunohistochemical analyses, the ITTC was positive for CD5, P63, CD117, and CK 5/6 and negative for thyroglobulin, calcitonin, and TTF 1. At the same time, PTC was positive for TTF 1 and thyroglobulin and negative for CD5, P63, and CK 5/6. The patient received postoperative radiotherapy and remained well with no evidence of recurrence during one month follow-up.

**Conclusion:**

Distinguishing ITTC from other thyroid malignancies before the surgery is challenging due to its non-specific presentations. Therefore, the diagnosis relies on postoperative studies, especially immunohistochemistry. The recommended treatment approach to improve survival in ITTC cases is total thyroidectomy combined with cervical lymph node dissection, followed by postoperative radiotherapy. The coexistence of ITTC and PTC may indicate the similarity in the underlying mechanisms of these tumors. However, further investigations are needed to understand this potential correlation.

## Introduction

Intrathyroid thymic carcinoma (ITTC) is a rare tumor of the thyroid gland, which accounts for less than 0.15% of all thyroid malignancies (1).

In 1985, for the first time, Miyauchi et al. described this disorder as intrathyroidal epithelial thymomas (2). Six years later, Chan and Rosai (3) categorized these neoplasms into four subgroups: ectopic cervical thymoma, ectopic hamartomatous thymoma, spindle epithelial tumor with thymic-like differentiation, and Thyroid carcinoma showing thymic-like differentiation (CASTLE). In 2017, the World Health Organization (WHO) defined the CASTLE subgroup as ITTC in the classification of endocrine organ tumors (4).

It has been proposed that ITTC originates from ectopic thymus tissues or remnants of branchial pouches (5, 6). ITTC exhibits structural resemblance to thymic tissue and expresses molecular markers typically found in thymic carcinomas and thymomas (7, 8). However, the mechanisms of pathogenesis remain unknown.

Due to the rare nature of this disorder, there are still no specific guidelines regarding the management of ITTC. Nevertheless, it is crucial to differentiate ITTC from other thyroid neoplasms as ITTC generally has a more favorable prognosis (9). The preoperative diagnosis of ITTC may be challenging due to the resemblance of its clinical manifestations and histological characteristics to other aggressive thyroid carcinomas. These similarities may lead to misdiagnosis and unnecessary aggressive treatments (2, 10). Hence, the definitive diagnosis of ITTC often relies on postsurgical pathological studies (11).

The coexistence of ITTC and papillary thyroid carcinoma (PTC) in the same thyroid gland is an extremely rare condition that has been reported only in a limited number of cases (12–17). As this coexistence may potentially affect the treatment strategies, prognosis, and outcomes of the ITTC, clinicians should be aware of this condition. In addition, the increasing number of cases reported with this simultaneous occurrence may provide possible clues to better understand the underlying pathogenesis of ITTC.

In this study, we present a case of the coexistence of ITTC and the follicular variant of PTC in a 26-year-old woman who underwent total thyroidectomy with postoperative radiotherapy and remained asymptomatic through the one- month follow-up.

## Case description

In December 2022, a 26-year-old Iranian woman was referred to the otolaryngology clinic of our institution, complaining of a growing neck mass, hoarseness, dysphagia, and nocturnal sweating over four months. Her family history and past medical history were unremarkable.

On examination, a palpable mass (approximately 4 × 4 cm) was located at the left thyroid lobe. The mass was mobile, non-tender, and hard in consistency. The rest of the physical examination findings were unremarkable.

The laboratory tests revealed no abnormalities, and her thyroid function test results were all in the normal range.

Neck ultrasonography showed the increased size of the thyroid gland (right lobe 50×19×17 mm and the left lobe 72×31×27 mm). Parenchymal echo of the isthmus and left thyroid lobe was decreased and heterogeneous, suggesting replacing the entire left lobe and the isthmus with a hypoechoic mass. Moreover, it revealed two hypoechoic nodules (sizes of 6 and 10 mm) with distinct boundaries in the right thyroid lobe. No calcification and lymphadenopathy were reported in the neck ultrasound.

Fine needle aspiration (FNA) cytology of the left lobe of the thyroid gland revealed colloid goiter without evidence of malignancy.

The patient underwent a total thyroidectomy. Intraoperatively, due to the firm consistency of the mass and suspicion of malignancy, the frozen section was performed. The frozen section analysis of the left lobe and isthmus was positive for malignancy. According to the lack of evidence regarding the lymph nodes’ involvement in ultrasound and benign FNA cytology results, only paratracheal lymph node dissection was performed for the patient, instead of complete neck dissection.

The postsurgical course was uneventful, and the patient was discharged on the third day following the operation.

Postoperative histopathological examinations of the surgical specimens obtained from surgery were performed. Gross examination showed the replacement of most of the left thyroid lobe and isthmus with a white multinodular mass, as well as a part of the right lobe with different tumors, including a white multinodular mass and two distinct white nodules. On microscopic examination of the same tumors in the left and right lobe by H and E staining, tumor cells had ill-defined cell borders with vesicular nuclei and distinct nucleoli arranged in well-demarcated lobular growth with fibrous bands separating variably sized solid islands and some lymphocytes between tumoral cells with focal necrosis. The immunohistochemical analyses of this tumor were positive for CD5, P63, CD117, CK 5/6, CKAE1/AE3, and Ki-67 labeling index (15-20%, hot spot) but negative for thyroglobulin, calcitonin, TTF 1, and TdT. Hence, based on the immunohistochemical results, this tumor was definitely diagnosed as ITTC.

The gross examination of two other smaller tumors, which were near the first tumor in the right lobe, showed two white cream nodules with sizes of 0.2 and 0.7 cm attached to the thyroid capsule. Microscopic examination of both nodules showed small to medium-sized follicular structures with nuclear grooves, intranuclear inclusions, and ground glass appearance. These tumors were diagnosed as follicular variant of papillary thyroid carcinoma according to the immunohistochemical analyses: negative for CD5, P63, CK 5/6, while positive for TTF 1, thyroglobulin, and CKAE1/AE3.

The excised paratracheal lymph nodes were tumor-free based on histopathological studies.

ITTC invaded the cervical muscles and fibrofatty tissue with no lymphovascular or perineural invasion, but the PTC tumor was localized into the thyroid gland and did not spread outside the thyroid capsule.

**Figure 1:**
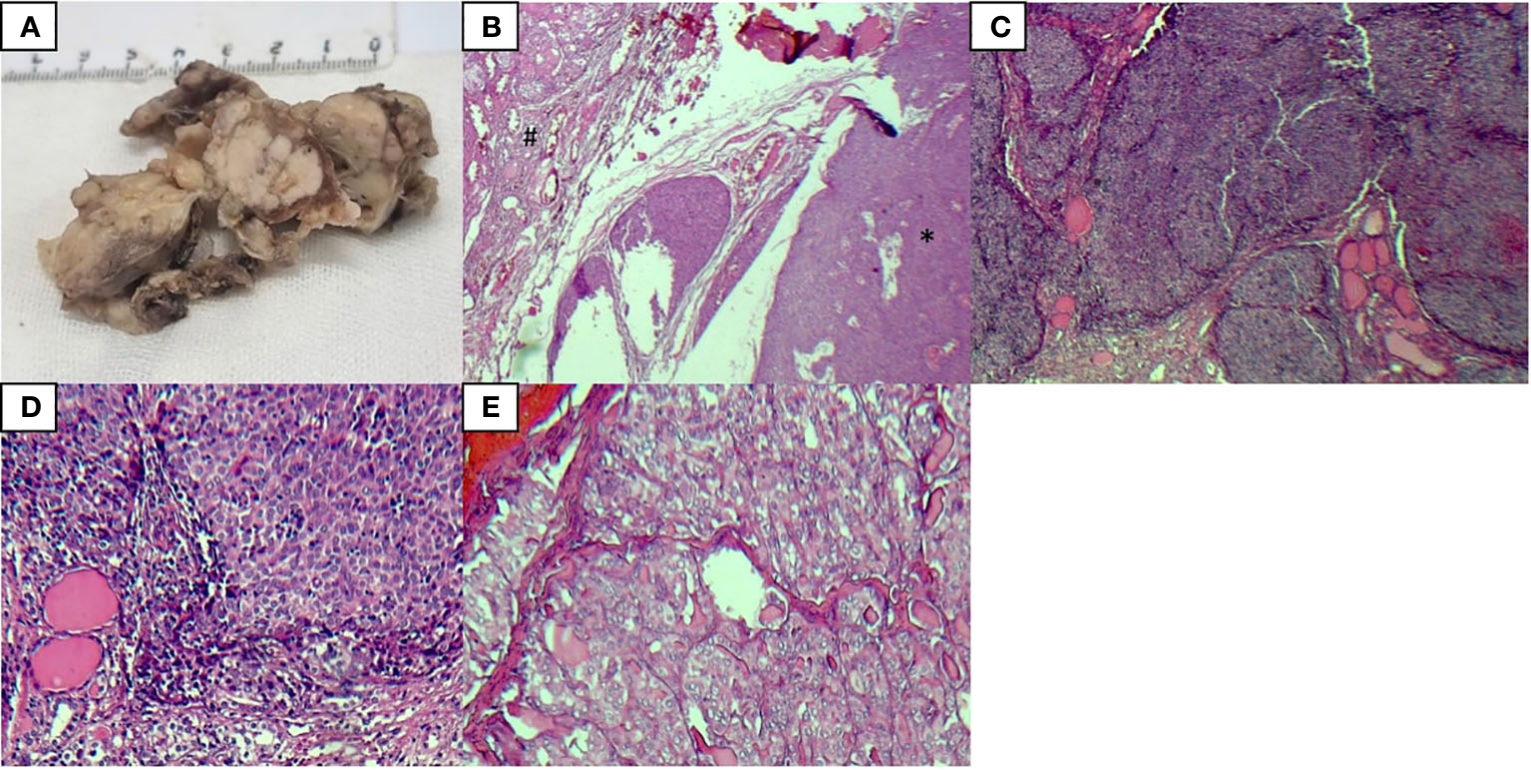
Pathological analysis results of tumors, ITTC (*), and PTC (#) **(A)** The gross examination of the left thyroid lobe showed a white multinodular mass invading most of the thyroid tissue with soft tissue extension, **(B)** H & E staining of ITTC and PTC, **(C, D)** H & E staining of ITTC, **(E)** H & E staining of PTC.

**Figure 2:**
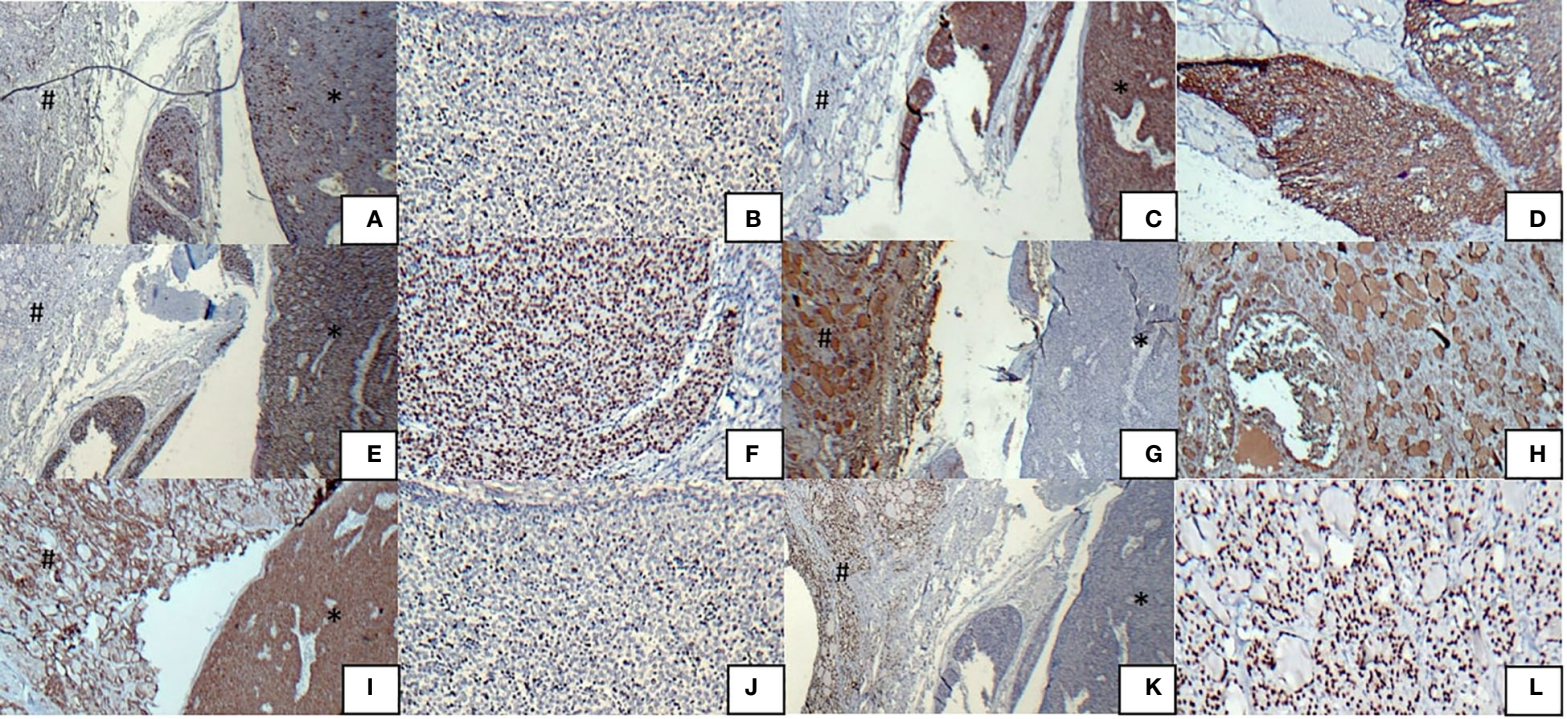
Immunohistochemical analysis results of tumors, ITTC (*) and PTC (#) **(A)** Immunostaining was positive for CD5 in ITTS, but negative in PTC, **(B)** CD5 positivity in ITTS, **(C)** Immunostaining was positive for CK 5/6 in ITTS, but negative in PTC, **(D)** CK 5/6 positivity in ITTS, **(E)** Immunostaining was positive for P63 in ITTS, but negative in PTC, **(F)** P63 positivity in ITTS, **(G)** Immunostaining was positive for thyroglobulin in PTC, but negative in ITTC, **(H)** Thyroglobulin positivity in PTC, **(I)** Immunostaining was positive for CKAE1/AE3 in both ITTS and PTC, **(J)** Ki67 positivity in 15-20% of ITTC cells, **(K)** Immunostaining was positive for TTF1 in PTC, but negative in ITTC, **(L)** TTF1 positivity in PTC.

The pathological and immunohistochemical analysis results of our case are shown in **Figures 1** and **2**, respectively.

The patient underwent postoperative radiotherapy with 60 Gy in 30 fractions over one month. She remained well during the one month of the follow-up period, with no tumor recurrence or metastasis on imaging studies.

## Discussion

Here, we presented a case of the coexistence of the ITTC and the follicular variant of PTC, which showed a favorable response to total thyroidectomy and postoperative radiotherapy. Also, we reviewed the clinical characteristics, treatment strategies, and outcomes of previously reported ITTC cases in the literature (**Supplementary Table S1**).

Among the 164 cases that were reviewed, the majority of patients were reported from East Asian countries, with China accounting for 74 cases (45%), followed by Japan with 43 cases (26%), South Korea with 8 cases (4%), and Taiwan with 6 cases (3%). However, there were also cases reported from other countries, albeit in smaller numbers (**Supplementary Table S1**). Notably, our case is the first reported case of ITTC from the Middle East, in an Iranian patient.

With a slight female predominance, ITTC usually involves middle-aged individuals in their fourth and fifth decades of life (9).

The majority of patients present with a painless, gradually enlarging neck mass. Patients may also experience hoarseness, dry cough, breath shortness, and swallowing difficulty, which may be related to the invasion of the tumor into surrounding soft tissues and local lymph nodes (18).

It is crucial to distinguish ITTC from other head and neck carcinomas because the prognosis and therapeutic approaches are different (19).

On ultrasound studies, ITTC may appear as a solid, lobulated, hypoechoic tumor with heterogeneous internal echoes with no evidence of calcification or cystic components (20).

Computed tomography (CT) scans typically show masses with unclear borders and soft tissue density, with no evidence of cystic changes and calcifications. These findings are inconsistent with other thyroid carcinomas, such as anaplastic and squamous-cell carcinomas. Metastatic lymph nodes also show similar features, including low-density masses with indistinct borders and slight enhancement following contrast administration (21). However, the presence of calcifications does not exclude the possibility of ITTC, as Dong et al. reported calcifications in the scans of three patients with ITTC (22).

Magnetic resonance imaging does not provide additional diagnostic benefits (21). ITTC has also been noted as a “cold nodule” on technetium single-photon emission computed tomography imaging, which is also commonly seen in other thyroid cancers (5).

According to the above, preoperative examinations do not yield a definitive diagnosis for ITTC. Therefore, the definite diagnosis mainly depends on postsurgical pathological examinations, particularly immunohistochemical studies (23).

While FNA biopsy plays a crucial role in diagnosing thyroid malignancies, it is unable to differentiate ITTC from less differentiated thyroid neoplasms, such as squamous cell carcinoma, poorly differentiated carcinoma, and anaplastic thyroid carcinoma (2, 10). However, needle biopsy may provide tissue samples with appropriate sizes for conducting immunohistochemical studies (19). In our case, FNA cytology did not diagnose the malignancy and was reported as negative for malignancy.

Microscopic examinations of ITTC often show squamoid, polygonal, or spindle-shaped tumor cells with oval-shaped nuclei, well-defined nucleoli, and eosinophilic cytoplasm. Also, the presence of prominent nucleoli with low mitotic counts is usually reported (5, 24, 25).

The immunohistochemical staining of ITTC typically reveals a strong positivity for CD5, cytokeratin, and p63 but negativity for thyroid tissue-related markers such as calcitonin, thyroglobulin, and TTF1 (18, 26). These findings align with the results of immunohistochemical analyses in our case.

Due to the rare nature of ITTC, there are currently no specific guidelines for the management of this condition. Surgery is usually recommended as the primary treatment option (11, 27). Patients with extrathyroidal extensions may have a higher susceptibility to lymph node metastasis. Therefore, prophylactic lymph node dissection might be a practical approach to reduce the local recurrences in these patients (11). According to the previous reports, patients who underwent total thyroidectomy combined with cervical lymph node dissection experienced desirable outcomes with a local recurrence rate of 14% and survival rate of 90% at five years and 82% at ten years (28).

Previous studies have shown patients with regionally invasive tumors and metastasis cervical lymph nodes who received radiotherapy experienced lower local recurrence rates (26). Additionally, the beneficial effects of radiotherapy have been shown in patients with no metastasis to the lymph nodes. This suggests that the use of radiotherapy may not be limited to cases with evidence of metastasis, and surgery combined with adjuvant radiotherapy is a promising therapeutic approach to improve the survival of ITTC (11).

Meanwhile, previous studies have shown that both the primary ITTC tumor and metastatic lesions exhibit hypermetabolic uptake of 18F-FDG on positron emission tomography (PET)-CT scans (27, 29). These findings may indicate the potential utility of PET-CT scans in staging and monitoring the ITTC response to postoperative radiotherapy. Unfortunately, due to the patient’s poor economic situation and the unavailability of PET-CT scan at our center, we were unable to perform this procedure for our patient. However, we recommend PET-CT scan studies for staging and monitoring of tumor in the management of patients with ITTC.

Chemotherapy has been proposed as another available modality for the treatment of ITTC. However, the effectiveness of chemotherapy is still under debate, and former investigations have failed to achieve a significant enhancement in the survival of patients (10, 11).

To the best of our knowledge, this is the eighth case of the concurrent occurrence of ITTC and PTC within the same thyroid gland (12–17). While a direct pathological association between ITTC and PTC has not been reported in the literature, some potential correlations, such as shared genetic alterations and environmental factors, may be involved in this coexistence. Previous studies have recommended the presence of TERT promoter mutations as a potential genetic factor related to cancer progression in ITTC (30). Similarly, these mutations have been reported in 4.7% of papillary thyroid microcarcinomas (31). Furthermore, it has been reported that some genetic alternations in ITTC are associated with the NF-kB signaling pathway (32), which plays a role in promoting PTC progression (33, 34). These shared genetic alterations and the concurrent occurrence of ITTC and PTC may indicate similarities in the underlying molecular mechanisms involved in the occurrence and development of these two cancers. However, it is crucial to acknowledge that these possible correlations are supported by limited evidence, and the coexistence of these tumors may be accidental. Further research is needed to understand the underlying pathological mechanisms of ITTC.

While our study reported the first case of ITTC from the Middle East and provided an updated review of previously reported cases, we acknowledge several limitations. Firstly, due to the lack of evidence of lymph node involvement in the radiologic studies and the benign results of the FNA, the diagnosis of malignancies was confirmed through postoperative histopathological studies, and the surgeon performed selective dissection of the paratracheal lymph nodes instead of complete neck dissection for our patient. However, we recommend considering a total thyroidectomy combined with complete neck dissection in the management of ITTC, to minimize the risk of potential recurrence. Secondly, in addition to our case, the coexistence of ITTC and PTC has been documented in seven previously reported cases (12–17). Nonetheless, the increasing number of reported cases with this simultaneous occurrence may provide novel insights into the underlying pathophysiological mechanisms of ITTC. Further biological studies in the future may enhance our understanding of this coexistence.

## Conclusion

The concurrent occurrence of ITTC and the follicular variant of PTC is an extremely rare condition that may indicate the similarity in these tumors’ molecular underlying pathogenic mechanisms. Since the non-specific presentation of ITTC makes it challenging to distinguish it from other thyroid malignancies, diagnosis is often based on postsurgical studies, especially immunohistochemistry. Total thyroidectomy combined with cervical lymph node dissection, followed by postoperative radiotherapy is the recommended treatment strategy to enhance the patients’ survival.

## Data availability statement

The original contributions presented in the study are included in the article/supplementary material, further inquiries can be directed to the corresponding author/s.

## Ethics statement

The studies involving humans were approved by Ethics committee of Shahid Sadoughi University of Medical Sciences, Yazd, Iran. The studies were conducted in accordance with the local legislation and institutional requirements. The participants provided their written informed consent to participate in this study. Written informed consent was obtained from the individual(s) for the publication of any potentially identifiable images or data included in this article.

## Author contributions

MV: Data curation, Investigation, Writing – original draft, Writing – review & editing. AAt: Data curation, Writing – original draft. MP: Data curation, Writing – original draft. AAl: Data curation, Writing – original draft. VZ: Investigation, Writing – review & editing. MB: Writing – original draft. MM: Data curation, Investigation, Project administration, Writing – original draft, Writing – review & editing. NZ: Investigation, Writing – review & editing.
