# Supplementary material for: Coexistence of intrathyroid thymic carcinoma and papillary thyroid carcinoma: a case report and literature review
Source: Front Oncol. 2024 May 3;14:1394020. doi: 10.3389/fonc.2024.1394020 (PMC11099278; doi:10.3389/fonc.2024.1394020)
Supplement: Supplementary file 1 [file Table_1.docx]

**Supplementary material:**

**Table S1. Characteristics and outcomes of reported ITTC cases in the literature.**

| **Case number** | **Study** | **Country** | **Age/Gender** | **Symptoms** | **Tumor location** | **Tumor size (cm)** | **Treatment** | **Outcome** |  |
| --- | --- | --- | --- | --- | --- | --- | --- | --- | --- |
|  | Abeni (1) | Italy | 26/M | Throat tightness -neck edema | Thyroid with bilateral cervical LN metastasis | N/d | TT + Bilateral functional lymphadenectomy + Chemotherapy + RT | Alive with NER at 3 Mon. |  |
|  | Abu-Salah (2) | United States | 31/F | Neck swelling - tenderness | Right thyroid with skeletal muscle and lymphovascular invasion | 3 | Right thyroidectomy + Central ND + RT | Alive with NER at 3 Mon. |  |
|  | Abuchaibe (3) | United States | 45/F | N/d | Inferior part of the left thyroid with lymphovascular invasion and extrathyroidal extension | 3.5 × 2.5 × 1.5 | TT | N/d |  |
|  | Ahuja (4) | Hong Kong | 67/F | Neck mass | Extra-thyroid, at carotid and posterior spaces | 5 × 7 × 9 | Resection + ND + RT | N/d |  |
|  | Alifano (5) | France | 63/F | Neck swelling | Tracheal cartilage, lower part of the left thyroid, left superior horn of the thymus, and sternothyroid muscle with metastases to the cervical LN | 6 | En bloc resection + RT | Alive with NER at 6 Mon. |  |
|  | Ardighieri (6) | Italy | 35/M | Neck mass | Sublingual gland with perineural infiltration and focal lymphovascular invasion | 1.3 × 1 × 0.7 | Sublingual gland excision + ND + RT | Alive with NER at 24 Mon. |  |
|  | Asa (7) | Canada | 35/M | Thyroid mass | Right thyroid | N/d | Sub-total thyroidectomy + Right RLN resection | Subglottic tumor recurrence at 9 years, treated with RT recurrence with extension into the trachea and right vocal paralysis  after 15 years, treated with surgery | |
|  | Biswas (8) | United Kingdom | 75/F | Neck mass | Right thyroid with bilateral cervical LN invasion | 3 × 3 | TT + Selective ND | Alive with metastasis (received RT) involving thoracic vertebrae at 18 Mon. | |
|  | Cappelli (9) | Italy | 73/M | Hoarseness -dyspnea | Right thyroid with right lateral cervical LN invasion and pleural metastasis | N/d | Thoracentesis + Chemotherapy | Died of disease at 2 Mon. | |
|  | Chan (10) | Taiwan | 54/M | Neck mass | Lower part of left thyroid and left upper mediastinum | 4.8 | Median sternotomy + Left thyroid lobectomy | Alive with NER  at 36 Mon. | |
|  | Chang (11) | South Korea | 34/F | Sore throat | Lower part of the right thyroid with extrathyroidal invasion, and regional LN metastasis | 3.3 | TT | Alive with NER  at 27 Mon. | |
|  | Chen (12) | China | 55/M | Asymptomatic | Lower part of the right thyroid with tracheoesophageal groove invasion and right posterior tracheal wall infiltration | 2.7 × 2.3 | TT + RT | N/d | |
|  | Cheon (13) | South Korea | 58/F | Hoarseness | Right thyroid extended to the right supraclavicular fossa with RLN invasion and neck LN metastasis | 4.2 | TT + Selective ND + Chemotherapy | Alive with disease and persisting right vocal cord paralysis at 48 Mon. | |
|  | Choi (14) | South Korea | 73/M | Hoarseness | Lower part of the left thyroid with perithyroid soft tissue invasion | 2.2 × 1.9 × 2.8 | TT + Central ND + RT | Alive with NER  at 72 Mon. | |
|  | Choi (15) | South Korea | 27/M | Neck mass | Both thyroid lobes and right submandibular area | 3.8 × 3.2 × 3.8 | Excision of the submandibular region mass along with six right level IIa LN+ Modified radical ND + Ipsilateral thyroid lobectomy | Alive with NER  at 36 Mon. | |
|  | Chow (16) | China | 43/M | Growing painful thyroid nodule | Thyroid, esophagus, left laryngeal nerve, cervical muscles, and trachea | N/d | Debulking surgery + RT | Alive with NER  at 72 Mon. | |
|  | Chow (16) | China | 49/M | Neck mass - upper airway obstruction symptoms | Thyroid, trachea and superior mediastinum | N/d | Debulking surgery + RT + Chemotherapy | Alive with NER  at 30 Mon. | |
|  | Chow (16) | China | 62/F | Hoarseness - dysphagia | Left thyroid, superior mediastinal and trachea | 3.8 | TT + Chemotherapy + RT | Alive with NER  at 21 Mon. | |
|  | Cui (17) | China | 41/F | Neck mass | Middle-lower region of the right thyroid | 1.6 × 1.1 × 1.3 | TT + Right central ND | Alive with NER at 48 Mon. | |
|  | Damiani (18) | Italy | 47/F | Neck mass - dyspnea -swallowing difficulty | Central part of thyroid, extending to tracheal wall | 5 | Tracheostomy + RT | Alive with NER  at 84 Mon. | |
|  | Dang (19) | Vietnam | 60/F | Neck mass - breath shortness | Left side of the anterior mediastinum and left cervical LN with extra-nodal extension into sternocleidomastoid muscle | 1.6 × 5.4 | Declined the treatment | Alive and stable at 15 Mon. | |
|  | Dong (20) | China | 52/M | Neck mass | Middle to lower part of the left thyroid extended to the substernal region with strap muscle, and trachea invasion | 5 | TT + Central ND | Alive with NER  at 81 Mon. | |
|  | Dong (20) | China | 61/F | Neck Mass -hoarseness | Left thyroid | 5 | TT + Central ND | Alive with NER  at 43 Mon. | |
|  | Dong (20) | China | 47/F | Neck mass | Lower part of the right thyroid, extended to the substernal region with strap muscle and carotid sheath invasion and LN metastasis | 4 | TT + Central ND + Bilateral modified ND | Recurrence after 26 Mon.,  subsequent operation, alive with NER at 35 Mon. | |
|  | Dong (20) | China | 54/F | Neck mass | Lower part of the right thyroid with strap muscle invasion | 2 | TT + Central ND | Alive with NER  at 29 Mon. | |
|  | Dong (20) | China | 57/M | Neck mass -hoarseness | Lower part of the right thyroid with strap muscle and RLN invasion | 3 | TT + Central ND | Alive with NER  at 26 Mon. | |
|  | Dong (20) | China | 50/M | Neck mass -hoarseness | Right thyroid with strap muscle invasion | 5 | TT + Central ND + Modified ND | Alive with NER  at 23 Mon. | |
|  | Dualim (21) | Malaysia | 58/M | Hoarseness -intermittent hemoptysis - breath shortness | Left thyroid with left retrosternal extension | 4.3 × 3.9 × 5.2 | Debulking surgery | Alive with NER  at 6 Mon. | |
|  | Ebina (22) | Japan | 63/M | Dyspnea - neck mass | Right thyroid with trachea and esophagus invasion | 5 | Total pharyngolaryngectomy + Near-total thyroidectomy + Bilateral ND + Cervical esophagus reconstruction + Chemoradiotherapy | Alive with NER at 120 Mon. | |
|  | Fung (23) | China | 49/M | Hoarseness - thyroid mass | Right thyroid with right tracheal wall and right RLN invasion | 4.4 × 3.5 × 6.0 | TT + Excision of one-third of the tracheal wall + Selective ND + RT | Alive with NER  at 18 Mon. | |
|  | Gao (24) | China | 61/F | Neck mass | Anterior neck with retrosternal extension | 5 | Right thyroid lobectomy + Chemoradiotherapy | Local recurrence at 20 Mon., treated with RT,  Local recurrence at 6 years, treated with curative wide local excision, central ND and postoperative RT,  A third local recurrence at 1 year later, treated with concurrent chemotherapy  and RT;  Died from acute respiratory distress at 1 Month after the initiation of treatment. | |
|  | Gao (24) | China | 48/M | Thyroid mass | Thyroid with left RLN, strap muscle, trachea, and esophagus invasion | N/d | TT + Central ND + RT | Alive with NER  at 24 Mon. | |
|  | Gao (24) | China | 67/F | Hoarseness - dysphagia | Left thyroid with superior mediastinal and tracheal invasion | 3.8 | TT + Central ND + Chemotherapy + RT | Alive with NER  at 48 Mon. | |
|  | Gaudreau (25) | Canada | 41/F | Neck mass | Lower part of the right thyroid | 2.8 × 3.5 × 3.6 | Hemi-thyroidectomy | N/d | |
|  | Geraci (26) | Italy | 63/M | Thyroid enlargement - dysphagia | Lower part of the both thyroid lobes | N/d | TT + RT | Alive with NER at 12 Mon. | |
|  | Gonzales (27) | Peru | 49/F | Neck mass | Left lateral wall of trachea | 6 | Partial tumor resection + RT | Alive with NER at 60 Mon. | |
|  | Hamada (28) | Japan | 63/M | Slowly expanding mass in the left parotid region | Left parotid gland | 2.8 × 2.3 | Resection +ND + RD | Alive with NER at 6 Mon. | |
|  | Huang (29) | China | 41/F | Neck mass | Lower part of the right thyroid | 4 × 3 | Thyroidectomy + Modified ND + Right sternothyroid muscle resection + Trans sternal lymphadenectomy + RT | Alive with NER at 12 Mon. | |
|  | Hanamura (30) | Japan | 46/M | Thyroid nodule | Lower part of the right thyroid  with central and right lateral neck LN metastases | 3.2 | TT + Modified radical ND + RT | Lung metastasis and tumor recurrence at 13 Mon., treated with chemotherapy; second tumor recurrence at 32 Mon., treated with chemotherapy | |
|  | Hsu (31) | Taiwan | 58/F | Hoarseness | Right paratracheal region | 2.0 × 2.5 | TT + Central ND + RT | N/d | |
|  | Hirokawa (32) | Japan | 44/M | N/d | lower part of the Right thyroid | 2.8 | N/d | Alive at 97 Mon. | |
|  | Hirokawa (32) | Japan | 29/F | N/d | Right thyroid | 5.8 | N/d | Alive at 272 Mon. | |
|  | Hirokawa (32) | Japan | 73/M | N/d | Lower part of the right thyroid | 2.7 | N/d | N/d | |
|  | Hirokawa (32) | Japan | 47/F | N/d | Left thyroid | 5.9 | N/d | Alive at 132 Mon. | |
|  | Hirokawa (32) | Japan | 25/F | N/d | Lower part of the right thyroid | 2.2 | N/d | Alive at 58 Mon. | |
|  | Ishikawa (33) | Japan | 23/F | Mass on left parotid gland | Left parotid gland | 2.3 × 3.6 × 3.4 | Superficial parotidectomy with peripheral LN dissection + RT | Alive with NER at 13 Mon. | |
|  | Jiang (34) | China | 40/M | Neck mass | Left thyroid with suprasternal fossae invasion | N/d | Radical resection + Chemoradiotherapy | Alive at 96 Mon. | |
|  | Jiang (34) | China | 52/M | Neck mass | Right thyroid with trachea, mediastinum  and carina invasion | N/d | Chemoradiotherapy | Lung metastasis at 60 Mon., treated with palliative chemotherapy followed by PD-1 inhibitor immunotherapy | |
|  | Jiang (34) | China | 56/M | Neck mass | Right thyroid with trachea invasion | N/d | Radical resection + Chemoradiotherapy | Alive with NER  at 130 Mon. | |
|  | Jiang (34) | China | 54/F | Neck mass | Left thyroid with esophagus, mediastinum, and vertebral front  fascia invasion | N/d | Palliative resection +  Chemoradiotherapy | Alive with NER at 42 Mon. | |
|  | Jiang (34) | China | 68/F | Neck mass | Left thyroid with esophagus, mediastinum, and common carotid artery invasion | N/d | Palliative resection +  Chemoradiotherapy | Alive with NER at 39 Mon. | |
|  | Jiang (34) | China | 49/F | Neck mass | Left thyroid with trachea and upper  esophagus invasion | N/d | Radical resection +  Chemoradiotherapy | Neck recurrence with lung and bone metastasis at 97 Mon., treated with palliative chemotherapy | |
|  | Jiang (34) | China | 45/M | Neck mass | Left thyroid with trachea, esophagus and vertebral front fascia invasion | N/d | Palliative resection +  Chemoradiotherapy | Lost the follow-up at 22 Mon. | |
|  | Jin (35) | China | 41/F | Neck mass | Lower part of the left thyroid | 1.8 × 1.6 | TT + Central ND | Alive with NER at follow-up | |
|  | Kałużna (36) | Poland | 70/F | Neck mass | Right thyroid | 2.5 × 2.5 × 3 | TT+ Neck lymphadenectomy | LN metastasis after two years, NER in subsequent follow ups | |
|  | Kakudo (37) | Japan | 59/M | Neck mass | Lower part of the left thyroid | 4 × 4 × 3.5 | Sub-total thyroidectomy + modified radical ND + RT | Pulmonary metastasis at 14 Mon., died  of disease at 17 Mon. | |
|  | Kimura (38) | Japan | 28/M | Neck mass | Lower part of the left thyroid | 3.0 × 2.0 | Left hemithyroidectomy + Central ND | Alive with NER at 120 Mon. | |
|  | Kimura (39) | Japan | 66/M | Hoarseness - cough | Lower part of the right thyroid with right RLN, the membranous trachea, the esophageal envelope, and cervical LN invasion | 1.8 × 2.4 × 2.6 | TT + Central and right ND + RT | Alive with NER at 60 Mon. | |
|  | Kovářová (40) | Czech Republic | 66/M | Hoarseness - dyspnea | Arising from the left but infiltrating both thyroid lobes | 4.0 × 5.5 × 6.0 | RT | Alive with NER at 24 Mon. | |
|  | Kuroki (41) | Japan | 66/M | Hoarseness - cough | Lower part of the left thyroid with tracheal invasion | 2.1 × 2.1 | TT + Bilateral LN dissection + Cervical tracheal resection + Tracheal reconstruction | Recurrence in the left neck and mediastinal LN at 35 Mon. treated with neck and mediastinal dissections.  Re-recurrence at 45 and 62 Mon. treated with surgical resections. Re-recurrence at 77 Mon. treated with concurrent chemoradiotherapy with cisplatin.  Alive with cancer at 90 Mon. after the first surgery | |
|  | Kusada (42) | Japan | 68/M | Right anterior neck swelling | Right thyroid with lung metastasis | 4.5 | Right thyroid lobectomy + Adjacent lymphadenectomy + External irradiation | Died of disease at 11 Mon. with lung metastasis | |
|  | Lakshmi (43) | India | 72/F | Supraclavicular swelling | Lower part of the left thyroid | 4.9 × 4.5 | Chemotherapy + Left modified radical ND + Left hemi-thyroidectomy + RT | Alive with NER at 96 Mon. | |
|  | Lee (44) | South Korea | 66/M | N/d | Lower part of the right thyroid | N/d | Total thyroidectomy + LN dissection + RT | N/d | |
|  | Li (45) | China | 52/M | Hoarseness | Right thyroid with tracheal extension | 3.8 | Surgery | Alive with NER at 11 Mon. | |
|  | Li (45) | China | 44/F | Asymptomatic | Right thyroid with esophageal extension and LN metastasis | 4.8 | Apatinib mesylate + Surgery | Alive with NER at 7 Mon. | |
|  | Li (45) | China | 53/F | Asymptomatic | Right thyroid with banded muscle extension and LN metastasis | 5.0 | Surgery + Postoperative RT | Alive with NER at 20 Mon. | |
|  | Li (45) | China | 66/F | Hoarseness - Cough | Right thyroid with banded muscle extension | 5.0 | Preoperative RT + Surgery | Alive with lung metastasis at 57 Mon. | |
|  | Li (45) | China | 37/F | Hoarseness | Left thyroid with tracheal extension and LN metastasis | 3.0 | Surgery + Postoperative chemoradiotherapy | Alive with NER at 95 Mon. | |
|  | Li (45) | China | 33/M | Asymptomatic | Right thyroid and LN metastasis | 2.5 | Surgery + Postoperative chemoradiotherapy | Alive with NER at 22 Mon. | |
|  | Li (45) | China | 64/M | Asymptomatic | Right thyroid with esophageal extension | 4.8 | Surgery | Alive with NER at 25 Mon. | |
|  | Li (45) | China | 49/F | Asymptomatic | Right thyroid with banded muscle extension | 1.8 | Surgery + Postoperative RT | Alive with NER at 12 Mon. | |
|  | Li (45) | China | 31/F | Hoarseness - Pain | Right thyroid with esophageal extension | 4.0 | Surgery + Postoperative chemoradiotherapy | Alive with NER at 91 Mon. | |
|  | Liu (46) | Taiwan | 67/F | Hoarseness - easy choking | Left lower neck compressing the trachea with tracheal wall invasion and focally infiltration to the thyroid | N/d | Partial tracheotomy + Tumor excision | Alive with NER at 12 Mon. | |
|  | Liu (47) | China | 32/F | N/d | Lower part of right thyroid with strap muscle invasion | 2 | Right thyroid lobectomy +  Right strap muscle  resection + ND + RT | Alive with NER at 2 Mon. | |
|  | Liu (47) | China | 62/M | N/d | Lower part of left thyroid with strap muscle, left internal jugular vein, and left RLN invasion | 5 | Left thyroid lobectomy + Left  strap muscle resection + Left  internal jugular vein and RLN  resection + Bilateral modified ND + RT | Alive with NER at 4 Mon. | |
|  | Liu (47) | China | 62/F | N/d | Isthmus and left thyroid with left internal jugular vein and left RLN invasion | 5 | TT +Left internal jugular  vein and RLN resection+ Bilateral modified ND + RT | Alive with NER at 4 Mon. | |
|  | Liu (47) | China | 45/M | N/d | Left thyroid with strap muscle invasion | 5 | Left thyroid lobectomy + Left  strap muscle resection + Left modified ND + RT | Alive with NER at 12 Mon. | |
|  | Liu (47) | China | 40/F | N/d | Lower part of the right thyroid with strap muscle invasion | 4 | Right thyroid lobectomy +  Right strap muscle resection + Right modified ND + RT | Alive with NER at 12 Mon. | |
|  | Liu (47) | China | 79/M | N/d | Lower part of the right thyroid and the upper part of the left thyroid | 3 | TT + Central ND | Alive with NER at 15 Mon. | |
|  | Liu (47) | China | 52/M | N/d | Lower part of the right thyroid | 4 | Right thyroid lobectomy +  Right selective ND + RT | Alive with NER at 27 Mon. | |
|  | Liu (47) | China | 73/M | N/d | Lower part of the right thyroid | 6 | Right thyroid lobectomy + Right modified ND + RT | Alive with NER at 45 Mon. | |
|  | Lominska (48) | United States | 60/M | Dysphonia - Bilateral upper extremity weakness - Fatigue | Left thyroid with lymphovascular and perineural invasion | 7 | TT + Central ND + RT + Chemotherapy | Alive with NER at 36 Mon. | |
|  | Lorenz (49) | Switzerland | 79/F | Progressive dyspnea - productive cough - poor appetite -weight loss | Right upper lobe of the lung with cardiophrenic, hilar, and mediastinal LN extension | 4.2 × 2.2× 2.8 | Left partial Pleurectomy + Talc pleurodesis + Pembrolizumab | No residual pleural effusion, alive with disease at 11 Mon. | |
|  | Luo (50) | Taiwan | 47/M | Neck mass | Extrathyroid, left parapharyngeal  space with left  carotid artery and  submandibular gland displacement | 5 × 5 × 4 | Left thyroid lobectomy + RT | Alive with NER at 21 Mon. | |
|  | Marini (51) | Greece | 76/M | Dyspnea - cough - dysphagia - cervical pain | Extending from sternoclavicular joints to the  upper borders of the thyroid gland surrounding the trachea | 4.76 × 4.01 | En bloc tumor resection + TT + Regional LN dissection + Tracheostomy | N/d | |
|  | Misfeld (52) | Australia | 68/M | Neck pain - dysphagia | Close to the thyroid cartilage and trachea,  surrounding the subclavian arteries, the right common carotid, and the brachiocephalic trunk. | 6.4 × 6.7 × 10.0 | Chemotherapy + Surgical resection + Subclavian arteries, brachiocephalic trunk, right carotid, and reconstruction | N/d | |
|  | Miyauchi (53) | Japan | 51/F | Neck mass | Lower part of the right thyroid | 4.0 × 3.5 | Right Thyroid lobectomy + ND | Alive with NER at 17 years | |
|  | Miyauchi (53) | Japan | 59/F | Cervical nodule | Middle to lower part of the left thyroid | 4.0 × 3.0 | Partial lobectomy | Local Recurrence at 17 years | |
|  | Miyauchi (53) | Japan | 47/F | Goiter - pressure  on neck | Left thyroid with left RLN invasion | 4.0 × 3.0 | Left thyroid lobectomy + Modified radical ND + Resection of the left RLN | Alive with NER at 14 years | |
|  | Miyauchi (53) | Japan | 65/F | Neck mass | Lower part of the left thyroid | N/d | Sub-total thyroidectomy + ND + RT | Alive with NER at 14 years | |
|  | Miyauchi (53) | Japan | 52/M | Hoarseness | Lower part of the left thyroid, fixed to the trachea | N/d | Sub-total thyroidectomy + ND + Tracheoplasty + RT | LN metastasis and recurrence in mediastinum at 6 years, treated with RT; died of disease at 8 years. | |
|  | Miyauchi (53) | Japan | 29/F | Neck mass | Middle to lower part of the right thyroid fixed to the trachea | N/d | Sub-total thyroidectomy + ND + Tracheoplasty + RT | LN metastasis and tumor recurrence in the lung at 36 Mon., treated with partial resection of the lung. Alive with pleural effusion at 8 years | |
|  | Mizukami (54) | Japan | 59/F | Neck mass | Middle to lower part of the right thyroid perithyroidal soft tissues invasion | 4.8 × 3 × 3 | Sub-total thyroidectomy + Bilateral ND | Alive with NER at 24 Mon. | |
|  | Mizukami (54) | Japan | 71/M | Neck mass | N/d | 7 × 8 × 3 | TT + Laryngotracheal resection + Radical ND | Died of local recurrence at 6 Mon. after surgery | |
|  | Morikawa (55) | Japan | 38/F | Neck mass | Left thyroid | 1.6 × 1.0 | Thyroid lobectomy +  LN dissection | Alive with NER at 36 Mon. | |
|  | Ng (56) | Hong Kong | 39/F | Neck discomfort | Lower part of the right thyroid | 2.5 × 2 × 1.5 | Right hemi-thyroidectomy | N/d | |
|  | Nogami (57) | Japan | 52/F | Neck mass - dyspnea - bloody sputum | Thyroid with tracheal invasion | 8 | Thyroidectomy + Complete resection + RT | Lung and bone recurrences at 11 Mon.,  treated with palliative RT to the bone metastasis and drainage of pleural effusion at 12 Mon. after surgery.  Died of pleural metastasis at 14 Mon. after the initial diagnosis | |
|  | Noh (58) | South Korea | 76/ M | N/d | Right thyroid with extraparenchymal extension | N/d | Surgical resection + Adjuvant RT | Alive with NER at 41 Mon. | |
|  | Noh (58) | South Korea | 54 /F | N/d | Left thyroid with extraparenchymal extension | N/d | Surgical resection + Adjuvant therapy | Alive with NER at 29 Mon. | |
|  | Noh (58) | South Korea | 59/ F | N/d | Left thyroid with extraparenchymal extension | N/d | Surgical resection + Adjuvant therapy | Alive with NER at 11 Mon. | |
|  | Okubo (59) | Japan | 56/M | Hoarseness | Middle to lower part of the left thyroid with laryngeal nerve and vascular invasion | 2.5 × 2.2 | Surgical resection | Alive with NER at 60 Mon. | |
|  | Okubo (59) | Japan | 68/M | Hoarseness | Lower part of the left thyroid with vascular and laryngeal nerve invasion | 3.0 × 2.1 | Hemi-thyroidectomy + Chemoradiotherapy | Mediastinum metastasis at 7 years, treated with surgery. No recurrence was observed 3 years after the second surgery. | |
|  | Okubo (59) | Japan | 52/F | N/d | Lower part of the left thyroid with vascular invasion | 1.2 × 1 | Hemithyroidectomy | Alive with NER at 8 Mon. | |
|  | Okuma (60) | Japan | 54/M | Neck mass - hoarseness | Left thyroid with superior mediastinum invasion and pulmonary, hepatic and cerebral metastases | 4.5 | Chemotherapy | Died of disease at 5 Mon. after the initiation of chemotherapy | |
|  | Pan (61) | China | 58/M | N/d | Middle to lower part of the left thyroid | 2.5 | Thyroid lobectomy + ND | Alive with NER at 36 Mon. | |
|  | Pan (61) | China | 69/M | N/d | Lower part of the left thyroid | 3 | Thyroid lobectomy + ND + RT | Alive with NER at 32 Mon. | |
|  | Pan (61) | China | 43/F | N/d | Left thyroid | 1 | Thyroid lobectomy + ND + RT | Alive with NER at 24 Mon. | |
|  | Pan (61) | China | 51/F | N/d | Right thyroid | 1.5 | Tumor excision | Alive with NER at 24 Mon. | |
|  | Pan (61) | China | 54/M | N/d | Left thyroid | 2.5 | Thyroid lobectomy + ND | Alive with NER at 15 Mon. | |
|  | Pan (61) | China | 45/M | N/d | Left thyroid | 10 | Thyroid lobectomy + ND + RT + Chemotherapy | Alive with NER at 18 Mon. | |
|  | Pan (61) | China | 48/F | N/d | Right thyroid | 3.5 | Thyroid lobectomy + ND | Alive with NER at 6 Mon. | |
|  | Pan (61) | China | 52/M | N/d | Middle to superior part of the left thyroid | 2 | Sub-total thyroidectomy + ND + RT | Alive with NER at 3 Mon. | |
|  | Pati l(62) | India | 34/F | Hoarseness | Lower part of thyroid with regional LN invasion | N/d | Right hemi-thyroidectomy + ND | Alive with NER at 12 Mon. | |
|  | Piacentini (63) | Italy. | 47/F | N/d | Left thyroid | 3.6 × 2.9 × 2.6 | TT + Central ND + RT | Alive with NER at 12 Mon. | |
|  | Rajeshwari (64) | India | 40/F | Neck mass | Inferior part of the right thyroid and the trachea | 4 × 3 | TT + Excision of the mass | N/d | |
|  | Stanciu (65) | Romania | 50/F | Dyspnea - dry cough - weight loss - poor appetite - fatigue - dysphagia - dysphonia | Lower part of the left thyroid with the trachea, esophagus, hyoid muscles, and left inferior jugular vein invasions | 3 × 3.5 × 3.7 | TT + Cervical lymphadenectomy + RT + Chemotherapy | Alive with NER at 6 and 24 Mon. | |
|  | Steger (66) | Germany | 62/F | Neck mass | Both thyroid lobes | 4 × 4 | TT + Central ND + Resection of the left RLN and the left internal jugular vein + Chemoradiotherapy | PulMonary metastases at 15 Mon. | |
|  | Shek (67) | China | 39/F | Neck mass | Lower part of the right thyroid | 2.4 × 2.0 × 2.0 | Resection of the isthmus and right thyroid | Alive with NER at 15 Mon. | |
|  | Solomon (68) | Romania | 63/M | Neck mass | Lower part of the left thyroid | 3 | Sub-total thyroidectomy + Central ND + Chemotherapy + RT | N/d | |
|  | Sun (69) | China | 56/M | N/d | Lower part of the right thyroid | 4.5 | Right thyroid lobectomy and central ND + RT | Alive with NER at 61 Mon. | |
|  | Sun (69) | China | 47/M | N/d | Lower part of the left thyroid | 4.0 | Mass excision + Left thyroid lobectomy + central ND + RT | Local recurrence at 22 Mon., treated with subsequent operation + chemotherapy; Alive with NER at 52 Mon. after subsequent operation | |
|  | Sun (69) | China | 25/M | N/d | Middle to lower part of the left thyroid | 6.0 | Left thyroid lobectomy + Tumor excision+ Central ND + RT | Local recurrence after 12 years, treated with the subsequent operation and RT; Alive with NER at 35 Mon. | |
|  | Sun (69) | China | 51/F | N/d | lower part of the right thyroid with regional LN invasion | 4.0 | Right thyroid lobectomy + Resection of the right RLN + Right ND + RT | Alive with NER at 34 Mon. | |
|  | Sun (69) | China | 56/F | N/d | Middle to lower part of the left thyroid | 4.0 | Left thyroid lobectomy + Central ND + RT | Alive with NER at 30 Mon. | |
|  | Sun (69) | China | 53/M | N/d | Lower part of the right thyroid | 3.0 | Right thyroid lobectomy + Central ND + RT | Alive with NER 24 Mon. | |
|  | Sun (69) | China | 45/F | N/d | Lower part of the left thyroid | 3.0 | Left thyroid lobectomy + Central ND + RT | Alive with NER at 12 Mon. | |
|  | Tai (70) | Taiwan | 34/M | Neck mass | Left thyroid | 4.3 × 4.4 × 3.6 | Left thyroid lobectomy + RT | Alive with NER at 20 Mon. | |
|  | Tran (71) | United States | 53/F | Hoarseness - right vocal cord paralysis - neck mass | Right thyroid with tracheal and esophageal invasion | N/d | TT+ Central ND + RT | N/d | |
|  | Tsutsui (72) | Japan | 75/M | Hoarseness | Lower part of the left thyroid with RLN, trachea, esophagus, jugular vein, and vagus nerve invasion | 3.5 | Lobectomy + Modified radical ND | Alive with NER at 113 Mon. | |
|  | Tsutsui (72) | Japan | 70/F | Neck mass | Lower part of the right thyroid with RLN, trachea, esophagus, jugular vein, and vagus nerve invasion | 3.5 | TT + Modified radical ND | Alive with NER at 67 Mon. | |
|  | Tsutsui (72) | Japan | 47/M | Hoarseness | Left thyroid with RLN, trachea and esophagus invasion and LN and lung metastasis | 7.1 | TT + Modified radical ND + RT | Alive with NER at 66 Mon. | |
|  | Tsutsui (72) | Japan | 48/F | Neck mass | lower part of the left thyroid with RLN, trachea, and esophagus invasion and LN metastasis | 2.2 | TT + Modified radical ND + RT | Alive with NER at 38 Mon. | |
|  | Tsutsui (72) | Japan | 70/F | Neck mass | Lower part of the right thyroid with RLN invasion, and LN metastasis | 2.5 | Lobectomy + RT | Alive with NER at 50 Mon. | |
|  | Tsutsui (72) | Japan | 58/F | Neck mass | Left thyroid | 6 | RT | Died of disease at 129 Mon. | |
|  | Uchiyama (73) | Japan | 58/M | Discomfort in the right ear | Deep lobe of the right parotid gland | 2.0 × 2.0 | Right deep lobe parotidectomy + ND | Alive with NER at 13 Mon. | |
|  | Wang (74) | China | 46/M | N/d | Lower part of the right thyroid | 2.5 | Surgery | Alive with NER at 39 Mon. | |
|  | Wang (74) | China | 30/F | N/d | Lower part of the right thyroid | 2.5 | Surgery | Alive with NER at 31 Mon. | |
|  | Wang (74) | China | 57/M | N/d | Middle to lower part of the right thyroid with tracheal wall invasion | 6.5 | Surgery + RT | Alive with NER at 6 Mon. | |
|  | Wang (74) | China | 46/F | N/d | Lower part of the right thyroid | 3 | Surgery + RT | Local recurrence at 22 Mon., treated with subsequent surgery and RT; Alive at 65 Mon. | |
|  | Wang (74) | China | 58/F | N/d | Lower part of the left thyroid | 4.5 | Surgery | Alive with NER at 5 Mon. | |
|  | Wang (74) | China | 62/F | N/d | Lower part of the left thyroid | 3.5 | Surgery | Local recurrence after 18.5 Mon., treated with surgery and RT, Alive at 37 Mon. | |
|  | Wang (74) | China | 54/M | N/d | Middle to lower part of the left thyroid | 6 | Surgery + RT | Alive with NER at 39 Mon. | |
|  | Wang (74) | China | 31/F | N/d | Lower part of the right thyroid | 5.5 | Surgery | Alive with NER at 22 Mon. | |
|  | Wang (74) | China | 42/F | N/d | Lower part of the left thyroid | 3 | Surgery + RT | Alive with NER at 43 Mon. | |
|  | Wang (74) | China | 53/M | N/d | Lower part of the left thyroid | 4.8 | Surgery + RT | Alive with NER at 98 Mon. | |
|  | Watanabe (75) | Japan | 32/F | Neck mass | Lower part of the left thyroid | N/d | TT + ND | Alive with local recurrence at 5 Mon. | |
|  | Wong (76) | Switzerland | 55/F | Right parotid mass | Right parotid gland with sternocleidomastoid muscle invasion and LN metastasis | 1.8 × 2.4 × 2.3 | Right total parotidectomy with facial nerve preservation + Ipsilateral selective ND + RT | Alive with NER at 12 Mon. | |
|  | Wu (77) | Taiwan | 67/F | Hoarseness -cough - swallowing difficulty - easy choking - weight loss | Left tracheal wall at the thoracic inlet | 4.0 × 3.8 × 3.6 | Tumor resection + RT | Alive with NER at 8 Mon. | |
|  | Wu | China | 44/M | Neck mass - Breathing difficulties | Right thyroid | 4.0 × 3.1 | Right thyroid lobectomy | Alive with NER at 24 Mon. | |
|  | Yamamoto (78) | Japan | 43/F | Neck mass | Lower part of left thyroid with mediastinum invasion | 3 | Tumorectomy | N/d | |
|  | Yamamoto (78) | Japan | 54/F | Cough - neck mass | Lower part of the right thyroid with superior and anterior mediastinum invasion | 4 | Sub-total thyroidectomy | N/d | |
|  | Yamamoto (78) | Japan | 60/M | Hoarseness | Right thyroid | 3 | Right hemi-thyroidectomy | N/d | |
|  | Yamamoto (79) | Japan | 31/F | Right parotid mass – Paralysis of the right facial nerve | Right parotid gland | 3.5 | Total parotidectomy + Unilateral ND + RT | Alive with NER at 18 Mon. | |
|  | Yamamoto (79) | Japan | 40/F | Pain and swelling in the left submandibular region | Left submandibular gland | 1.7 | Submandibular gland resection + ND | Regional LN metastasis at 4 Mon., treated with chemotherapy; Lung metastasis at 4 years, treated with RT, chemotherapy + immunotherapy; Rib bone metastasis at 6 years, treated with RT; Alive with disease at 65 Mon. | |
|  | Yamazaki (80) | Japan | 62/M | Cough with sputum - neck mass | Lower part of the left thyroid with esophagus invasion and LN metastasis | N/d | Total pharyngo-laryngo-esophagectomy + TT + Reconstruction with a free jejunal graft | Alive with NER at 5 Mon. | |
|  | Yerly (81) | Switzerland | 58/M | Dysphonia - dysphagia | Lateral middle part of the left thyroid | 3.2 × 2.7 × 2.0 | Left thyroid lobectomy + lumpectomy + partial tracheal resection + RT | Alive with NER at 22 Mon. | |
|  | Yoneda (82) | Japan | 54/M | Hoarseness - breath shortness | Right thyroid with trachea invasion | 3.5 × 3.5 × 3.2 | TT + Internal jugular vein and segmental trachea resection + Reconstruction of the common carotid artery | Alive with NER at 24 Mon. | |
|  | Youens (83) | United States | 52/F | Neck swelling - intermittent neck pain | Left thyroid with left RLN, extra-laryngeal soft tissue, and tracheal cartilage invasion | 3.8 | Thyroidectomy + Primary tracheal resection + Left paratracheal ND + RT | Alive with NER at 5 Mon. | |
|  | Yuan (84) | China | 38/F | N/d | Lower part of the left thyroid | 3.0 × 2.5 | Thyroidectomy + Functional ND + RT | Tumor recurrence at 15 years, treated with RT and chemotherapy but progressed over next 5 years. | |
|  | Zhao (85) | China | 41/F | Neck mass | Lower part of the left thyroid | 3.0 × 2.0 | TT + Central ND | Alive with NER at 36 Mon. | |
|  | Zhao (85) | China | 39/M | Neck mass | Right thyroid | 4.6 × 2.6 × 2.2 | Lobectomy + Isthmectomy + Central ND | Alive with NER at 12 Mon. | |
|  | Zhang (86) | China | 37/F | Neck mass | Lower part of the right thyroid | 1 × 1 × 0.8 | Right thyroid lobectomy | Alive with NER at 6 Mon. | |
|  | Zhang (86) | China | 38/M | Hoarseness | Lower part of the right thyroid | 3.8 × 3 × 2.5 | Right thyroid lobectomy + Modified radical ND | Alive with NER at 26 Mon. | |
|  | Zhang (86) | China | 29/F | Neck mass | Isthmus and middle to lower part of the right thyroid with surrounding soft tissue invasion | 6 × 3 × 3 | TT + Radical ND | Alive with NER at 42 Mon. | |

LN: lymph nodes, N/d: not defined, NER: no evidence of recurrence, Mon.: months, RT: radiotherapy, TT: total thyroidectomy, RLN: recurrent laryngeal nerve, ND: neck dissection

**References:**

1. Abeni C, Ogliosi C, Rota L, Bertocchi P, Huscher A, Savelli G, et al. Thyroid carcinoma showing thymus-like differentiation: Case presentation of a young man. World J Clin Oncol. 2014;5(5):1117-20.

2. Abu-Salah AK, Segura S, Mesa H. Cytomorphologic findings of thyroid carcinoma showing thymus-like (CASTLE) differentiation: A case report. American Journal of Clinical Pathology. 2021;156:S42-S.

3. Abuchaibe C, Tourtelot J. A CASE REPORT OF THYROID CARCINOMA SHOWING THYMUS LIKE DIFFERENTIATION (CASTLE). Endocrine Practice. 2016;22:260.

4. Ahuja A, Chan E, Allen P, Lau K, King W, Metreweli C. Carcinoma showing thymiclike differentiation (CASTLE tumor). American journal of neuroradiology. 1998;19(7):1225-8.

5. Alifano M, Boudaya MS, Dinu C, Kadiri H, Regnard J-F. Carcinoma showing thymus-like elements invading the trachea. The Journal of Thoracic and Cardiovascular Surgery. 2006;132(1):191-2.

6. Ardighieri L, Tomasoni M, Battocchio S, Facchetti F, Maroldi R, Nicolai P, et al. Carcinoma Showing Thymus-Like Differentiation (CASTLE) Arising in the Sublingual Gland. Int J Surg Pathol. 2021;29(3):301-7.

7. Asa SL, Dardick I, Van Nostrand AP, Bailey DJ, Gullane PJ. Primary thyroid thymoma: a distinct clinicopathologic entity. Human pathology. 1988;19(12):1463-7.

8. Biswas D, Karsai L, Atkin S, England R. Management of carcinoma showing thymus-like element. The Journal of Laryngology & Otology. 2010;124(11):1242-4.

9. Cappelli C, Tironi A, Marchetti GP, Pirola I, De Martino E, Delbarba A, et al. Aggressive thyroid carcinoma showing thymic-like differentiation (CASTLE): case report and review of the literature. Endocr J. 2008;55(4):685-90.

10. Chan L-P, Chiang F-Y, Lee K-W, Kuo W-R. Carcinoma showing thymus-like differentiation (CASTLE) of thyroid: a case report and literature review. The Kaohsiung Journal of Medical Sciences. 2008;24(11):591-7.

11. Chang S, Joo M, Kim H. Cytologic Findings of Thyroid Carcinoma Showing Thymus-like Differentiation: A Case Report. Korean J Pathol. 2012;46(3):302-5.

12. Chen L, Chen X, Zhang H, Feng L, Zhu R. Carcinoma of the thyroid gland showing thymic-like elements: hypofunctioning nodule accumulating (99m)Tc-MIBI and (18)F-FDG. Hell J Nucl Med. 2011;14(2):190-1.

13. Cheon T, Song YJ, Kim JI, Cha HJ, Han MW. Aggressive thyroid carcinoma showing thymus-like differentiation (CASTLE) with lung metastasis and carotid artery invasion. Ear, Nose & Throat Journal. 2019;98(9):557-9.

14. Choi HG, Kim CS, Min SK, Park B. Carcinoma Showing Thymus-like Differentiation (CASTLE) with Non-Recurrent Laryngeal Nerve: A Case Report. Journal of Korean Thyroid Association. 2014;7(1):88-91.

15. Choi KY, Kwon MJ, Ahn HK, Kim JH, Lee DJ. Extrathyroid carcinoma showing thymus-like differentiation (CASTLE): a new case report and review of the therapeutic role of neck dissection and radiotherapy. World J Surg Oncol. 2014;12:247.

16. Chow SM, Chan JKC, Tse LLY, Tang DLC, Ho CM, Law SCK. Carcinoma showing thymus-like element (CASTLE) of thyroid: Combined modality treatment in 3 patients with locally advanced disease. European Journal of Surgical Oncology. 2007;33(1):83-5.

17. Cui A, Du Y, Hou C, Zhang L, Sun L, He H. A role of postoperative radiation therapy in completely resected early stage intrathyroid thymic carcinoma: a case report and literature review of the diagnosis and treatment. Frontiers in Oncology. 2023;13.

18. Damiani S, Filotico M, Eusebi V. Carcinoma of the thyroid showing thymoma-like features. Virchows Archiv A. 1991;418(5):463-6.

19. Dang N, Son L, Hong N, Nhung N, Tung N, Quang L. Recurrence of carcinoma showing thymus-like differentiation (CASTLE) involving the thyroid gland. Thyroid Research. 2021;14(1):1-6.

20. Dong W, Zhang P, Li J, He L, Wang Z, Zhang T, et al. Outcome of Thyroid Carcinoma Showing Thymus-Like Differentiation in Patients Undergoing Radical Resection. World J Surg. 2018;42(6):1754-61.

21. Dualim DM, Loo GH, Suhaimi SNA, Latar NHM, Muhammad R, Abd Shukor N. The ‘CASTLE’tumour: an extremely rare presentation of a thyroid malignancy. A case report. Annals of Medicine and Surgery. 2019;44:57-61.

22. Ebina A, Sugitani I, Motoi N. Intrathyroidal Epithelial Thymoma: Carcinoma Showing Thymus-like Differentiation Mimicking Squamous Cell Carcinoma of the Thyroid. Journal of Nippon Medical School. 2015;82(1):5-6.

23. Fung ACH, Tsang JS, Lang BHH. Thyroid Carcinoma Showing Thymus-Like Differentiation (CASTLE) with Tracheal Invasion: A Case Report. Am J Case Rep. 2019;20:1845-51.

24. Gao R, Jia X, Ji T, Feng J, Yang A, Zhang G. Management and Prognostic Factors for Thyroid Carcinoma Showing Thymus-Like Elements (CASTLE): A Case Series Study. Front Oncol. 2018;8:477.

25. Gaudreau A, Belisle A, Ayad T. An unusual neck tumor. European Annals of Otorhinolaryngology, Head and Neck Diseases. 2018;135(2):145-6.

26. Geraci G, Lo Nigro C, Sciuto A, Modica R, Francesco C, Sciumè C, et al. Carcinoma showing thymus-like differentiation (CASTLE): a case report. Ann Ital Chir. 2013;84(1):77-80.

27. Gonzales-Laguado E, Guerra-Miller H, Garcia-Ruiz L, Luna-Abanto J. Intrathyroidal thymic carcinoma misdiagnosed as a medullary thyroid carcinoma. Cirugia Y Cirujanos. 2020;88:35-8.

28. Hamada M, Miyama Y, Matsumura S, Shintani‐Domoto Y, Urano M, Yasuda M. Carcinoma showing thymus‐like elements (CASTLE) with amyloid deposition in the parotid gland. Pathology International. 2024.

29. Huang C, Wang L, Wang Y, Yang X, Li Q. Carcinoma showing thymus-like differentiation of the thyroid (CASTLE). Pathol Res Pract. 2013;209(10):662-5.

30. Hanamura T, Ito K, Uehara T, Fukushima T, Sasaki S, Koizumi T. Chemosensitivity in Carcinoma Showing Thymus-Like Differentiation: A Case Report and Review of the Literature. Thyroid. 2015;25(8):969-72.

31. Hsu Y-C, Hsueh C, Lin W-N, Tsai T-Y, Hung S-Y, Lu Y-A. Carcinoma Showing Thymus-like Differentiation (CASTLE) with Synchronous Papillary Thyroid Carcinoma: A Case Report and Review. Ear, Nose & Throat Journal. 2021:01455613211060167.

32. Hirokawa M, Miyauchi A, Minato H, Yokoyama S, Kuma S, Kojima M. Intrathyroidal epithelial thymoma/carcinoma showing thymus-like differentiation; comparison with thymic lymphoepithelioma-like carcinoma and a possibility of development from a multipotential stem cell. Apmis. 2013;121(6):523-30.

33. Ishikawa T, Ogawa T, Nakanome A, Yamauchi Y, Usubuchi H, Shiihara M, et al. Whole exome sequencing and establishment of an organoid culture of the carcinoma showing thymus-like differentiation (CASTLE) of the parotid gland. Virchows Archiv. 2021;478(6):1149-59.

34. Jiang L, Zheng WH, Chen C. Genomic variation associated with carcinoma showing thymus-like elements (CASTLE) in thyroid gland. Laryngoscope Investig Otolaryngol. 2022;7(3):894-900.

35. Jin X, Huang Z, Guo P, Yuan R. TOETVA: a single surgeon’s learning curve and a case report of CASTLE thyroid tumor. Langenbeck's Archives of Surgery. 2023;408(1):398.

36. Kałużna M, Janicki A, Sygut J, Marszałek A, Ziemnicka K, Ruchała M, editors. Patient with thyroid carcinoma showing thymus-like differentiation: a case study. Endocrine Abstracts; 2017: Bioscientifica.

37. Kakudo K, Bai YH, Ozaki T, Homma K, Ito Y, Miyauchi A. Intrathyroid epithelial thymoma (ITET) and carcinoma showing thymus-like differentiation (CASTLE). CD5-positive neoplasms mimicking squamous cell carcinoma of the thyroid. Histology and Histopathology. 2013;28(5):543-56.

38. Kimura E, Enomoto K, Kono M, Tamagawa S, Takeda S, Kumashiro N, et al. A Rare Case of Thyroid Carcinoma Showing Thymus-Like Differentiation in a Young Adult. Case Rep Oncol. 2021;14(1):671-5.

39. Kimura T, Enomoto K, Kono M, Hiraoka M, Takeda S, Kumashiro N, et al. A case of concurrent occurrence of carcinoma showing thymus-like differentiation and follicular variant of papillary thyroid cancer in the same thyroid. Journal of Surgical Case Reports. 2022;2022(1):rjab570.

40. Kovarova P, Vojtisek R, Krcma M, Daum O, Baxa J, Sukovska E, et al. Inoperable CASTLE of the thyroid gland treated with radical radiotherapy with complete remission. Strahlentherapie Und Onkologie. 2021;197(9):847-53.

41. Kuroki M, Shibata H, Iinuma R, Okuda H, Ohashi T, Ogawa T, et al. A Case of Thyroid Carcinoma Showing Thymus-Like Differentiation With Breast Cancer Susceptibility Gene 2 Mutation: A Case Report and Literature Review. Cureus. 2022;14(10).

42. Kusada N, Hara Y, Kobayashi S, Tang WH, Nakamura Y, Kakudo K, et al. A case of aggressive carcinoma showing thymus-like differentiation with distant metastases. Thyroid. 2005;15(12):1383-8.

43. Lakshmi J, Ullattil PK, Sidhique SK, Sreedevi M, LS MR. Carcinoma showing thymus-like differentiation (CASTLE)–A Rare Extra-Thyroid Disease.

44. Lee JH, Baek JM, Son BS, Park IY, Sung KY. Synchronous Carcinoma Showing Thymus-like Elements (CASTLE) of the Thyroid Combined with Rectal Carcinoma: Report of a Case and Review of the Literature. Korean Journal of Endocrine Surgery. 2008;8(4):266-8.

45. Li J, Xiang R, Li Y, Liao Q, Liu Y. Intrathyroid thymic carcinoma: clinicopathological features and whole exome sequencing analysis. Virchows Arch. 2023;482(5):813-22.

46. Liu S-M, Lee W-H. Carcinoma showing thymus-like differentiation (CASTLE): report of a case specified in the cytomorphology. J Formos Med Assoc. 2016;115(5):377-79.

47. Liu Z, Teng X-Y, Sun D-X, Xu W-X, Sun S-L. Clinical analysis of thyroid carcinoma showing thymus-like differentiation: report of 8 cases. International surgery. 2013;98(2):95-100.

48. Lominska C, Estes CF, Neupane PC, Shnayder Y, TenNapel MJ, O'Neil MF. CASTLE Thyroid Tumor: A Case Report and Literature Review. Front Oncol. 2017;7:207.

49. Lorenz L, von Rappard J, Arnold W, Mutter N, Schirp U, Scherr A, et al. Pembrolizumab in a Patient With a Metastatic CASTLE Tumor of the Parotid. Front Oncol. 2019;9:734.

50. Luo CM, Hsueh C, Chen TM. Extrathyroid carcinoma showing thymus-like differentiation (CASTLE) tumor--a new case report and review of literature. Head Neck. 2005;27(10):927-33.

51. Marini A, Kanakis M, Valakis K, Laschos N, Chorti M, Lioulias A. Thyroid Carcinoma Showing Thymic-Like Differentiation Causing Fracture of the Trachea. Case Rep Med. 2016;2016:7962385.

52. Misfeld M, Borger MA, Steinert F. Resection of a Tumor With Thymic-like Differentiation and Reconstruction of the Innominate Artery. Ann Thorac Surg. 2021;112(2):e99-e100.

53. Miyauchi A, Kuma K, Matsuzuka F, Matsubayashi S, Kobayashi A, Tamai H, et al. Intrathyroidal epithelial thymoma: an entity distinct from squamous cell carcinoma of the thyroid. World journal of surgery. 1985;9:128-34.

54. Mizukami Y, Kurumaya H, Yamada T, Minato H, Nonomura A, Noguchi M, et al. Thymic carcinoma involving the thyroid gland: Report of two cases. Human Pathology. 1995;26(5):576-9.

55. Morikawa Y, Ishihara Y, Kawano I, Matsuura N, Kaname A, Kakudo K. Cystic squamous cell carcinoma of the thyroid: A possible new subgroup of intrathyroidal epithelial thymoma. Endocrine Pathology. 1995;6:77-81.

56. Ng WK, Collins RJ, Shek WH, Ng IO. Cytologic Diagnosis of "CASTLE" of thyroid gland: report of a case with histologic correlation. Diagn Cytopathol. 1996;15(3):224-7.

57. Nogami T, Taira N, Toyooka S, Tanaka T, Mizoo T, Iwamoto T, et al. A case of carcinoma showing thymus-like differentiation with a rapidly lethal course. Case Rep Oncol. 2014;7(3):840-4.

58. Noh JM, Ha SY, Ahn YC, Oh D, Seol SW, Oh YL, et al. Potential Role of Adjuvant Radiation Therapy in Cervical Thymic Neoplasm Involving Thyroid Gland or Neck. Cancer Res Treat. 2015;47(3):436-40.

59. Okubo Y, Sakai M, Yamazaki H, Sugawara Y, Samejima J, Yoshioka E, et al. Histopathological study of carcinoma showing thymus-like differentiation (CASTLE). The Malaysian Journal of Pathology. 2020;42(2):259-65.

60. Okuma Y, Hosomi Y, Orimo K, Arai T, Hishima T. Aggressive course of CASTLE (carcinoma showing thymus-like differentiation) with distant metastasis and temporary activity of cisplatin and irinotecan. Head and Neck Oncology. 2013;5(3).

61. Pan Y, Zhao X, Yang J, Deng J, Zhan Z, Luo Y, et al. Absence of gene mutations in KIT-positive carcinoma showing thymus-like elements of the thyroid. Hum Pathol. 2012;43(3):350-5.

62. Patil VR, Patel K, Muzumdar G. Carcinoma showing thymus like differentiation (CASTLE)-A rare thyroid neoplasm. Thyroid Research and Practice. 2016;13(2):71.

63. Piacentini MG, Romano F, De Fina S, Sartori P, Leone EB, Rubino B, et al. Carcinoma of the neck showing thymic-like elements (CASTLE): report of a case and review of the literature. Int J Surg Pathol. 2006;14(2):171-5.

64. Rajeshwari M, Singh V, Nambirajan A, Mridha AR, Jain D. Carcinoma showing thymus like elements: Report of a case with EGFR T790M mutation. Diagn Cytopathol. 2018;46(5):413-8.

65. Stanciu M, Ristea RP, Popescu M, Vasile CM, Popa FL. Thyroid Carcinoma Showing Thymus-like Differentiation (CASTLE): A Case Report. Life (Basel). 2022;12(9).

66. Steger CM, Von Frankenberg M, Kahlert C, Mechtersheimer G, Steiner H, Schirmacher P, et al. CASTLE tumour of the neck: A rare location of a malignant tumour of the thymus. BMJ Case Reports. 2009.

67. Shek TW, Luk IS, Ng IO, Lo CY. Lymphoepithelioma-like carcinoma of the thyroid gland: lack of evidence of association with Epstein-Barr virus. Hum Pathol. 1996;27(8):851-3.

68. Solomon E, Solomon RG, Grigorovici M, Simon I, Preda C, editors. Carcinoma showing thymus-like differentiation (CASTLE)-a case report. Endocrine Abstracts; 2019: Bioscientifica.

69. Sun T, Wang Z, Wang J, Wu Y, Li D, Ying H. Outcome of radical resection and postoperative radiotherapy for thyroid carcinoma showing thymus-like differentiation. World J Surg. 2011;35(8):1840-6.

70. Tai CM, Liang CW, Chang TC. Intrathyroidal thymic carcinoma: a case report. J Formos Med Assoc. 2003;102(2):109-12.

71. Tran J, Zafereo M. Segmental tracheal resection (nine rings) and reconstruction for carcinoma showing thymus-like differentiation (CASTLE) of the thyroid. Head and Neck-Journal for the Sciences and Specialties of the Head and Neck. 2019;41(9):3478-81.

72. Tsutsui H, Hoshi M, Kubota M, Suzuki A, Nakamura N, Usuda J, et al. Management of thyroid carcinoma showing thymus-like differentiation (CASTLE) invading the trachea. Surgery today. 2013;43(11):1261-8.

73. Uchiyama T, Terada C, Tachibana Y, Nishiura H, Takeda M, Fujii T, et al. Carcinoma showing thymus‐like differentiation of the parotid gland: The brief report of cytomorphology and review of the literature. Diagnostic Cytopathology. 2023;51(3):E98-E104.

74. Wang Y-f, Liu B, Fan X-s, Rao Q, Xu Y, Xia Q-y, et al. Thyroid carcinoma showing thymus-like elements: a clinicopathologic, immunohistochemical, ultrastructural, and molecular analysis. American Journal of Clinical Pathology. 2015;143(2):223-33.

75. Watanabe I, Tezuka F, Yamaguchi M, Sagawa J, Kaise N. Thymic carcinoma of the thyroid. Pathol Int. 1996;46(6):450-6.

76. Wong EHC, Tetter N, Tzankov A, Muller L. CASTLE tumor of the parotid: First documented case, literature review, and genetic analysis of the cancer. Head Neck. 2018;40(1):E1-e4.

77. Wu M-H, Wu H-Y. Thyroid carcinoma showing thymus-like differentiation with tracheal invasion. Asian Cardiovascular and Thoracic Annals. 2016;24(9):878-80.

78. Yamamoto Y, Yamada K, Motoi N, Fujiwara Y, Toda K, Sugitani I, et al. Sonographic findings in three cases of carcinoma showing thymus-like differentiation. J Clin Ultrasound. 2013;41(9):574-8.

79. Yamamoto H, Kusafuka K, Nozaki Y, Iwasaki T, Nogami M, Hongo T, et al. Carcinoma showing thymus-like differentiation (CASTLE) of the salivary gland: Report of 2 cases of a hitherto under-recognized extrathyroid counterpart. Pathol Res Pract. 2021;227:153646.

80. Yamazaki M, Fujii S, Daiko H, Hayashi R, Ochiai A. Carcinoma showing thymus-like differentiation (CASTLE) with neuroendocrine differentiation. Pathol Int. 2008;58(12):775-9.

81. Yerly S, Lobrinus J-A, Bongiovanni M, Becker M, Zare M, Granger P, et al. A carcinoma showing thymus-like elements of the thyroid arising in close association with solid cell nests: evidence for a precursor lesion? Thyroid. 2013;23(4):511-6.

82. Yoneda K, Matsui O, Kobayashi T, Gabata T, Minato H, Hirokawa M. CT and MRI findings of carcinoma showing thymus-like differentiation. Radiat Med. 2005;23(6):451-5.

83. Youens KE, Bean SM, Dodd LG, Jones CK. Thyroid carcinoma showing thymus-like differentiation (CASTLE): case report with cytomorphology and review of the literature. Diagn Cytopathol. 2011;39(3):204-9.

84. Yuan Y, Ke C, Zhang G, Zhang J, Li Q. Case report and literature review: thyroid carcinoma showing intrathyroid thymic carcinoma. Front Oncol. 2022;12:923683.

85. Zhao Q, Bian X. Two cases of concurrent carcinoma showing thymus-like differentiation (CASTLE) coexisting with papillary thyroid carcinoma. J Surg Case Rep. 2023;2023(9):rjad527.

86. Zhang G, Liu X, Huang W, Li X, Johnstone M, Deng Y, et al. Carcinoma showing thymus-like elements of the thyroid gland: report of three cases including one case with breast cancer history. Pathology & Oncology Research. 2015;21(1):45-51.
